# Dynamics of Antibacterial Drone Establishment in Staphylococcus aureus: Unexpected Effects of Antibiotic Resistance Genes

**DOI:** 10.1128/mBio.02083-21

**Published:** 2021-11-16

**Authors:** Neha Dhasmana, Geeta Ram, Kathleen N. McAllister, Yulia Chupalova, Peter Lopez, Hope F. Ross, Richard P. Novick

**Affiliations:** a Department of Microbiology, New York University School of Medicine, New York, New York, USA; b Cytometry and Cell Sorting Laboratory, New York University School of Medicine, New York, New York, USA; University of Nebraska-Lincoln

**Keywords:** antibacterial drone (ABD), *Staphylococcus aureus*, tetracycline resistance, transduction, CRISPR

## Abstract

The antibacterial drone (ABD) system is based on repurposing the phage-inducible staphylococcal pathogenicity islands (SaPIs) for use as antibacterial agents that are indifferent to antibiotic resistance. The ABDs were constructed by inserting *tetM* for tetracycline resistance (Tc^r^) selection, replacing the SaPI virulence genes with bactericidal or bacteriostatic genes such as CRISPR/*cas9/agrA*, whose expression kills by double-strand cleavage of *agrA*, or CRISPR/*dcas9/agrP_2_P_3_*, whose expression blocks the target organism’s virulence. ABD DNA is packaged in phage-like particles that attack their staphylococcal targets *in vivo* as well as *in vitro*. We determine ABD titers by transfer frequency, enumerate surviving cells as a function of multiplicity, and analyze the fate of ABD DNA with green fluorescent protein. An initial study revealed surprisingly that many more cells were killed by the ABD than were measured by transduction. Our study of this phenomenon has revealed several important features of the ABD system: (i) a significant number of entering ABD DNA molecules do not go on to establish stable transductants (i.e., are abortive); (ii) ABD cargo genes are expressed immediately following entry, even by the abortive ABDs; (iii) immediate plating on Tc-containing agar seriously underestimates particle numbers, partly owing to Tc inhibition of protein synthesis; (iv) replacement of *tetM* with *cadA* (conferring resistance to CdCl_2_) provides more accurate particle enumeration; (v) ABDs expressing CRISPR/*cas9/agrA* kill ∼99.99% of infected cells and provide the most accurate measurement of particle numbers as well as proof of principle for the system; and (vi) surprisingly, TetM interferes with stable establishment of ABD DNA independently of Tc^r^.

## INTRODUCTION

**FIG 1:**
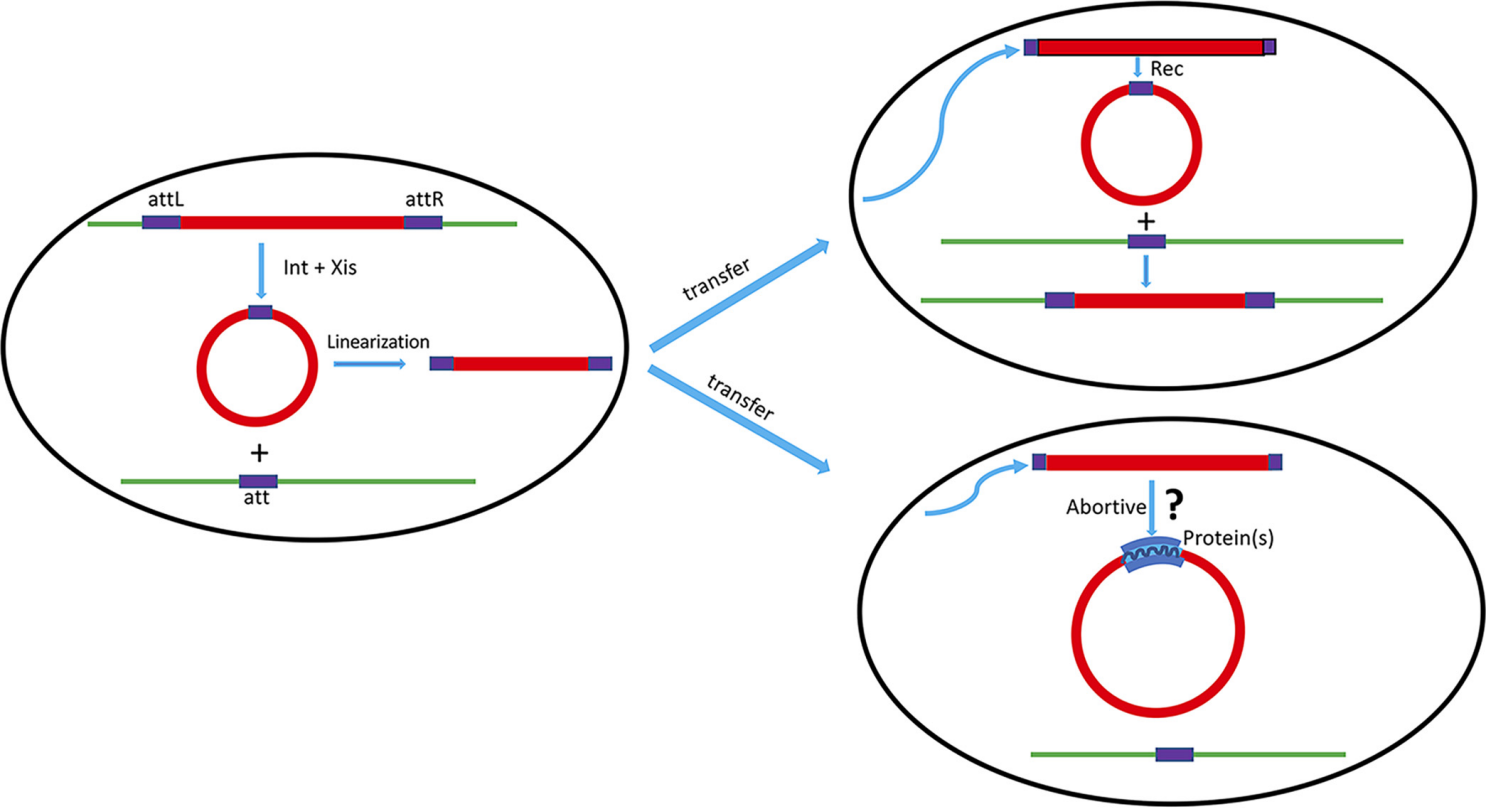
Transfer of integrated mobile element. (Left) Integrated element is excised and circularized, leaving an empty *att* site. It is then linearized and transferred to a recipient cell, where it either recircularizes and integrates into its *att* site by the Campbell mechanism or fails to integrate and becomes a linearly inherited abortive unit, which may be circularized by means of cellular or phage-encoded proteins, but its ends are not covalently joined.

Intercell transfer of integrating mobile genetic elements classically occurs by excision in circular form, linearization for transfer, recircularization in the recipient organism, and then integration into the chromosome or a plasmid by the classical Campbell mechanism (1) (Fig. 1). While the process in the donor cell seems clear, that in the recipient is less so. Considering *pac* phages (2) and phage-like elements such as the staphylococcal pathogenicity islands (SaPIs) (3), circularization involves a recombinase acting on paired redundant ends or on a multimer. This recombinase can be encoded by the element (4) or by the recipient chromosome, and this is often unclear; elements of the SaPI type are regulated by a master repressor, which may prevent establishment in a fraction of events. The details of this process are of more than academic interest, as they may significantly impact the horizontal transfer of mobile genetic elements and, in particular, the staphylococcal pathogenicity island (SaPI)-based antibacterial agents, briefly outlined below.

We have recently initiated the development of a novel antibacterial therapeutic platform utilizing repurposed 15-kb SaPIs as vectors for the delivery of bactericidal or -static genes (5). These engineered islands, packaged as phage-like particles, are known as antibacterial drones (ABDs) and are produced in very large numbers following mitomycin C induction of a c-resident helper prophage. We have deleted the helper phage small terminase (*terS*), required for packaging of phage DNA, but not SaPI/ABD DNA, and the SaPI capsid morphogenesis genes, required for the formation of small SaPI particles (6), resulting in the packaging of exclusively SaPI/ABD DNA in full-sized phage-like particles with a DNA capacity of 45 kb, of which >30 are available for cloning. As the DNA is packaged by the headful mechanism (2) from postreplicative concatemers (3), each particle will contain between 1 and 4 copies of the element depending on the length of the cargo DNA that has been added to the initial 12-kb backbone element, ABD2001.

To enable genetic selection, we inserted a tetracycline (7) resistance marker (*tetM*) so that ABD particle numbers could be measured by enumerating the number of tetracycline-resistant (Tc^r^) colonies when a suitable recipient strain was mixed with a sample of ABD particles and the mixture spread on a plate containing Tc. In an early study of SaPI biology (3), we observed that in the absence of a helper phage, the incoming linear, *tetM*-marked, SaPI DNA does not replicate and does not circularize and integrate for at least an hour following entry for reasons that will become clear later but eventually generates Tc^r^ colonies as expected.

The first ABDs incorporated two different CRISPR derivatives, one with *cas9* and a spacer targeting *agrA*, a highly conserved but nonessential chromosomal gene, and the second with *dcas9* and a spacer targeting the regulatory region of the *agr* locus (5).

With CRISPR/*cas9* targeting a chromosomal gene, we confirm that expression of *cas9* is lethal because *cas9* makes a double-stranded cut in the chromosomal protospacer, which is not repairable, since staphylococci, like most bacteria, do not have efficient nonhomologous end joining (8). Because, as currently configured, the ABD does not replicate and cannot spread, this means that a single ABD particle will kill a single cell. The killing titer is measured by infecting a specific number of cells with a specific number of particles and enumerating the surviving fraction. This number is inevitably an underestimate, because some 20 or 30% of staphylococci exist as clumps of 2 or 3 cells, and a clump will survive a lethal hit to one of its cells. A comparison of killing and transducing titers, enabled by using ABD2003 with target cells either containing or lacking the *agrA* protospacer, revealed, surprisingly, that the killing titer was much greater than the particle titer determined by Tc^r^ transduction. Investigation of the disparity between transduction titer and killing titer has led to a series of important findings that are especially relevant to ABD development and potential utility as well as to the biology of mobile genetic elements (MGE) in general.

## RESULTS

### Dynamics of tetracycline resistance.

**FIG 2:**
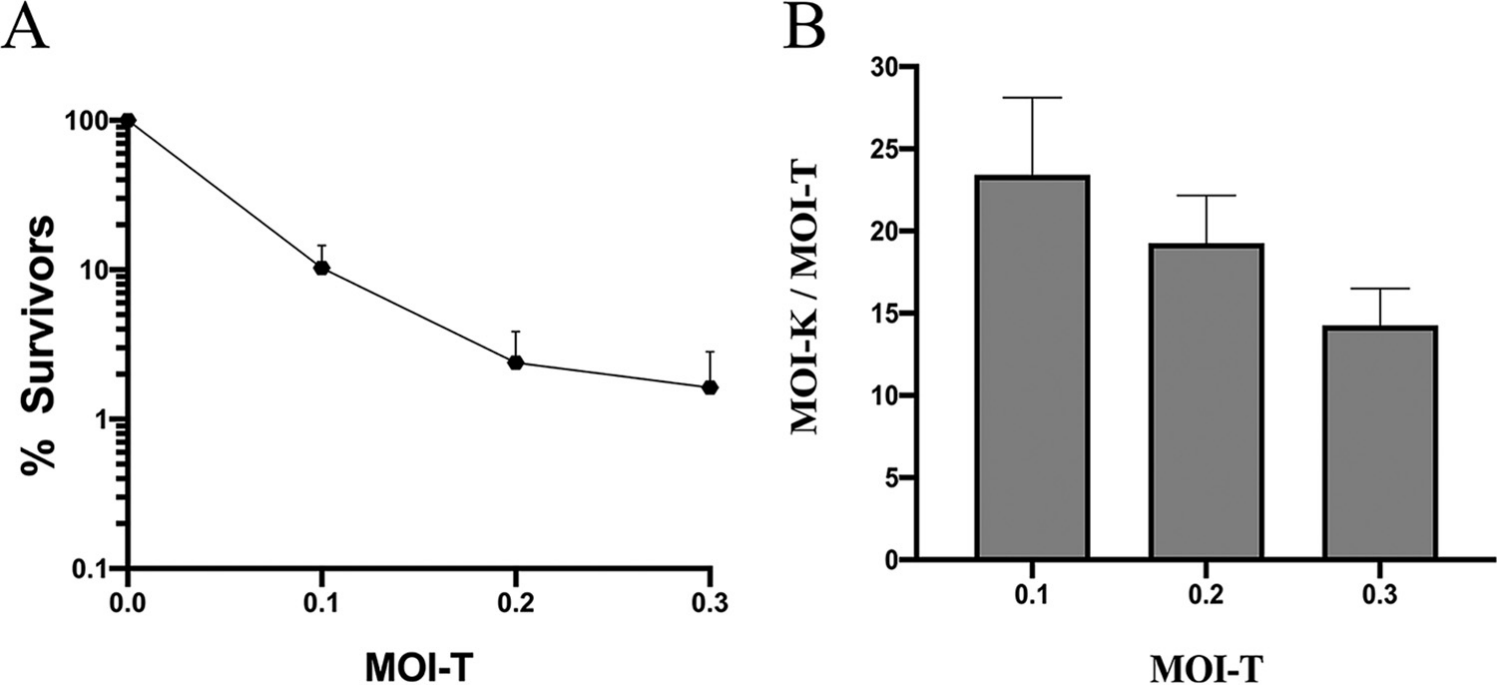
Killing versus transduction by ABD2003. (A) Killing titers were determined by mixing different numbers of ABD particles with a constant number of RN3 cells and then plating on TSB agar for survivors. Transduction titer was confirmed by mixing ABD particles with RN12414 (RN3Δ*agr*::*cadA*) and plating on TSB Tc5. Graphs represent data from three independent experiments. (B) Graph represent ratios of killing MOI (MOI-K) versus MOI calculated using Tc^r^. MOI-K were calculated by the Poisson formula for surviving fractions in panel A.

**FIG 3:**
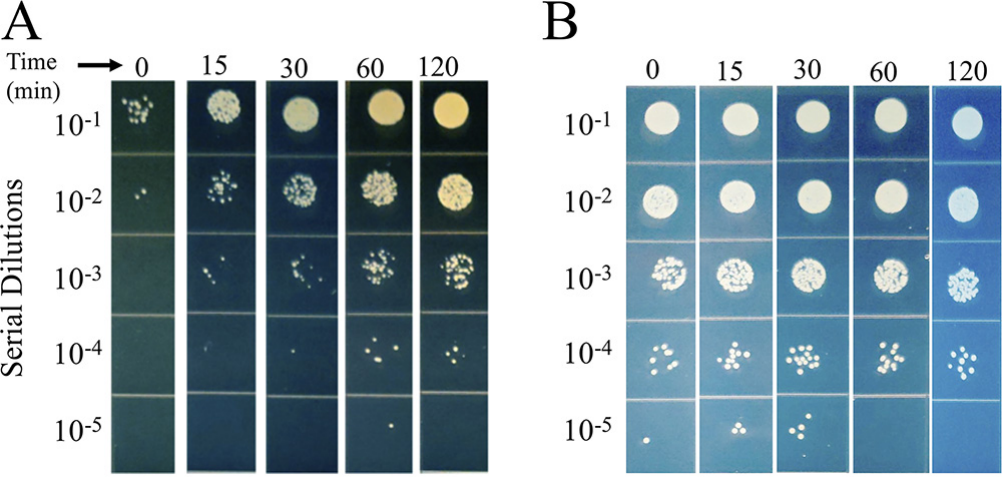
Effect of preincubation of transductants in the absence of Tc or CdCl_2_. (A) RN3 Δ*agrA* cells in phage buffer were infected with ABD2003 (Tc^r^) at an MOI-T of 0.3 and held at room temperature. Samples were removed at specific time intervals at 0, 15, 30, 60, and 120 min, followed by dilution and spot-plating on TSB Tc5. Plates were incubated at 37°C overnight. (B) RN3 cells were infected with ABD2030 (*cadA*-tagged ABD2001) at an MOI-C of 0.3. The mixture was held at room temperature. Samples were removed at specific time intervals of 0, 15, 30, 60, and 120 min followed by dilution and spot plating on GL-Cd100. The plates were incubated at 37°C overnight.

Tetracycline resistance (Tc^r^) has been effectively used as a selective marker in bacterial genetics for many years, generally scored by plating gene transfer mixtures directly on Tc-containing agar, even though Tc blocks protein synthesis. In a previous study (5), repeated here in more detail (Fig. 2), we compared bacterial killing by ABD2003, which contains CRISPR/*cas9/agrA*, with transduction for Tc^r^ with a recipient strain lacking the protospacer. For the killing assay, we mixed different numbers of ABD particles with a fixed number of cells and plated for survivors. We found the numbers of survivors to be much smaller (and, therefore, the killing titers to be much higher) than expected on the basis of particle numbers determined by transduction for Tc^r^. Using the Poisson formula to calculate the actual multiplicities of infection based on surviving cell numbers, we found that the true multiplicities were between 5- and 23-fold greater than expected on the basis of Tc^r^ titers, where the lower the MOI, the greater the difference. Since the entering ABD2003 DNA needs only to express a single gene in order to kill a cell while it must circularize and integrate, at the very least, in order to generate a transductant, it is not surprising that the killing titer is a much better indicator of the true particle number than is the transduction frequency; the inverse relation between transduction and killing frequencies suggests that the greater the number of infecting particles, the greater the chance of a successful transduction. This result suggested a solution for the above-described paradox, namely, that *tetM* is expressed slowly following ABD entry, with only a fraction of potential transductants becoming resistant during the 20 to 30 min between infection and plating, and that the greater the number of particles in a single cell, the greater the chance that Tc^r^ will be sufficiently expressed before plating. Even with several particles in a cell, full expression takes considerably longer. This interpretation predicts that if Tc were present at the time of infection and thereafter, no transductants would appear. This prediction was tested in the experiment shown in Fig. 3, in which Tc was either present throughout, or the transductants were incubated for various times in a nongrowth medium (phage buffer) in the absence of Tc before plating. As predicted, no transductants were obtained when cells and particles were mixed in the presence of Tc, and when the transduction mixture was incubated in the absence of Tc, the number of transductants increased steadily for 2 h. In order to maximize the transduction yield, one could simply allow expression for 2 h in the absence of drug before challenging with Tc. An alternative is to maintain the transductants in the presence of the inducer, anhydrotetracycline (9). In any case, the number of transductants was never greater than ∼50% of the killing titer.

### ABD establishment kinetics.

**FIG 4:**
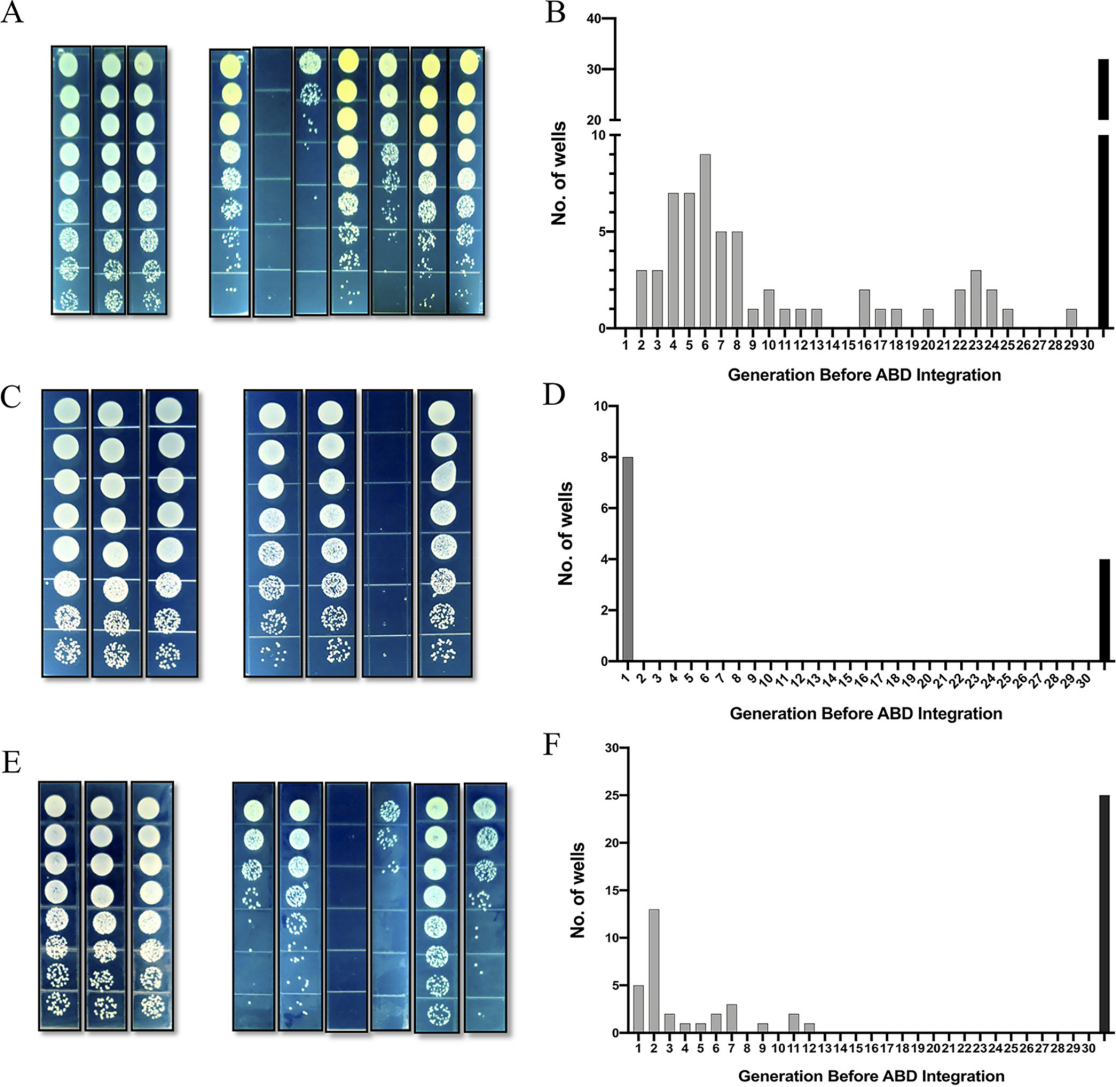
Establishment (integration) of ABD DNA in single cells. (A) The culture from each well of the 96-well plate was serially diluted (1:5 series), and 10 μl of each dilution was spot plated on TSB and TSB Tc5. The Tc^r^ fractions were used to calculate the number of generations before ABD integration in each well. (B) Number of wells showing ABD integration after each generation. Rightmost bar represents wells with no transductants. (C) Similar sorting experiment with ABD2034, labeled with *cadA* rather than *tetM*. Cells were plated on GL and GL-Cd100. (D) Number of wells showing ABD integration in each generation. Rightmost bar represents wells with no transductants (as in panel B). (E) Similar sorting experiment with ABD2034 and RN3 containing pCN36 (*tetM*). Cells were plated on GL and GL-Cd100. (F) Number of wells showing ABD integration in each generation. Rightmost bar represents wells with no transductants (as in panel B).

To investigate this seemingly strange result, we evaluated the ABD establishment process by a cell-sorting protocol in which individual cells are isolated immediately following receipt of an ABD DNA molecule (see Fig. S1 in the supplemental material). Here, using ABD2031, a derivative of ABD2001 to which had been cloned a green fluorescent protein (GFP) determinant, we mixed cells and GFP-labeled ABD particles at an MOI-T of about 0.3 (MOI-T refers to particle titers measured by Tc^r^ and MOI-C to those measured by CdCl_2_ resistance [Cd^r^]), held the mixture at 37°C for 30 min to allow maximum GFP expression, and then sorted the mixture with gating on GFP. A typical sort result is shown in Fig. S2. GFP-positive cells were then placed in a microtiter plate, one cell per well (10), containing TSB, and incubated overnight. Since the ABD DNA does not replicate autonomously (3), presumably owing to the early expression of the master repressor, *stl* (11), the ABD DNA would be inherited linearly until it integrated; thereafter, its population would increase in parallel with the rest of the population (ABD positives and negatives grow at the same rate). The result of this experiment was unexpected and rather dramatic, as shown in Fig. 4. Although most wells contained saturated bacterial cultures with about 10^9^ CFU, representing 30 generations of growth from a single cell, the number of ABD positives varied from zero in about 1/3 of the wells to about 5 × 10^8^ in a few, and the rest in between, meaning that the ABD either never integrated or integrated anywhere between 1 and about 28 generations after entry. In Fig. 4, the number of wells is plotted against the number of generations before ABD integration. These results suggest a time-dependent probability of integration such that the population of GFP^+^ Tc^r^ cells in any well is an indication of how many generations the initial cell had undergone before the ABD finally integrated. Remarkably, even with the most efficient recipient strain, e.g., NCTC8325, there was a considerable number of wells showing full bacterial growth but no transduced cells. This number corresponds to the irreducible ∼2-fold discrepancy between killing and transduction noted above, suggesting that there is a fraction of incoming ABD DNAs that never establish, which is similar to abortive transduction (12). The fate of the ABD DNA in these has not yet been determined. Note that this result explains the above-mentioned earlier finding of a >1-h delay in circularization/integration of incoming *tetM*-marked SaPI molecules.

10.1128/mBio.02083-21.1 FIG S1 Strategy used to study ABD integration using single cell sorting with FACS. NCTC8325 cells were infected with GFP-labeled ABD2031 (Tc^r^) or GFP-labeled ABD2034 (Cd^r^). The infected cells were incubated for 30 min for GFP expression and single GFP^+^ cells were sorted by FACS. These single GFP^+^ cells were placed in each well of 96-well plate and incubated at 37°C overnight. The generation before ABD integration was calculated using the standard curve. Download FIG S1, PDF file, 0.3 MB. Copyright © 2021 Dhasmana et al. 2021 Dhasmana et al. https://creativecommons.org/licenses/by/4.0/ This content is distributed under the terms of the Creative Commons Attribution 4.0 International license.

10.1128/mBio.02083-21.2 FIG S2 Gating strategy for isolation of ABD-infected cells. RN3 cells infected by ABD2031 or ABD2034 were sorted by FACS, with gating on GFP. (A) Uninfected cells were used to determine the specific gate (P1) for GFP-positive cells. (B) ABD2031 (Tc^r^)- or ABD2034 (Cd^r^)-infected cells were sorted using a GFP filter in the FACS Aria cell sorter (70-mm nozzle). Single GFP-positive cells from the P1 gate were placed in the wells of a 96-well microtiter plate containing 150 μl of TSB and incubated at 37°C overnight. Download FIG S2, PDF file, 0.1 MB. Copyright © 2021 Dhasmana et al. 2021 Dhasmana et al. https://creativecommons.org/licenses/by/4.0/ This content is distributed under the terms of the Creative Commons Attribution 4.0 International license.

### Kinetics of ABD cargo gene expression and killing.

The concordance of killing titer with Poisson-based calculations of MOI suggests that ABD cargo genes are expressed and functional before the cell division following ABD entry and that enough copies of a lethal cargo are produced to ensure the killing of any postdivision cell that has not inherited an abortive ABD molecule. To confirm this, we measured the kinetics of expression of *gfp* using the *gfp*-tagged ABD2034. (The results are shown in Fig. S3.) As can be seen, a GFP signal is detected within 30 min of ABD infection and increases linearly thereafter. With the *gfp* gene used in this experiment, 1,000 GFP molecules per cell are required for a signal to be detected. Since, following ABD DNA entry, the gene must be transcribed and many copies of the protein synthesized, it is suggested that a 30-min lag is consistent with immediate initiation of expression. The probability that an ABD2003-infected cell will be killed was determined by an experiment in which ABD2003-infected *agrA^+^* and *agrA* mutant cells, using a low MOI to ensure single infections, were plated for transduction. The transduction frequencies differed by 10^4^-fold, with the only *agrA^+^* transductants representing CRISPR-resistant mutants. In other words, every *agrA^+^* cell infected with ABD2003 and destined to become a transductant was killed, with the exception of CRISPR-resistant mutants. This experiment, however, cannot address the question of whether ABDs destined to be abortive transductants always kill the infected cell.

10.1128/mBio.02083-21.3 FIG S3 GFP expression profile after ABD2031 infection in S. aureus NCTC8325. The infection experiment was set up with GFP-labeled ABD at an MOI of 1 and incubated at room temperature for 20 min. CYGP medium was added to the mix and incubated at 37°C (static). The samples were taken out at the indicated time points, washed three times, and resuspended in PBS. The fluorescence readings were taken at 480/509 nm using a BioTek Synergy H1 hybrid plate reader. Download FIG S3, PDF file, 0.1 MB. Copyright © 2021 Dhasmana et al. 2021 Dhasmana et al. https://creativecommons.org/licenses/by/4.0/ This content is distributed under the terms of the Creative Commons Attribution 4.0 International license.

### ABD establishment but not killing is marker dependent.

To determine whether the observed integration pattern was marker specific, we replaced *tetM* with *cadA*, which confers CdCl_2_ resistance (Cd^r^). Resistance to cadmium salts, first identified as one of a set of heavy metal resistances (*cadA*) carried by staphylococcal plasmid pI258 (13), has become a very useful selective marker for staphylococcal genetics. As *cadA* resistance couples hydrolysis of ATP with active export of cadmium ions from recipient cells (14), cadmium does not block protein synthesis and therefore should be expressed in the presence of an inhibitory CdCl_2_ concentration.

**FIG 5:**
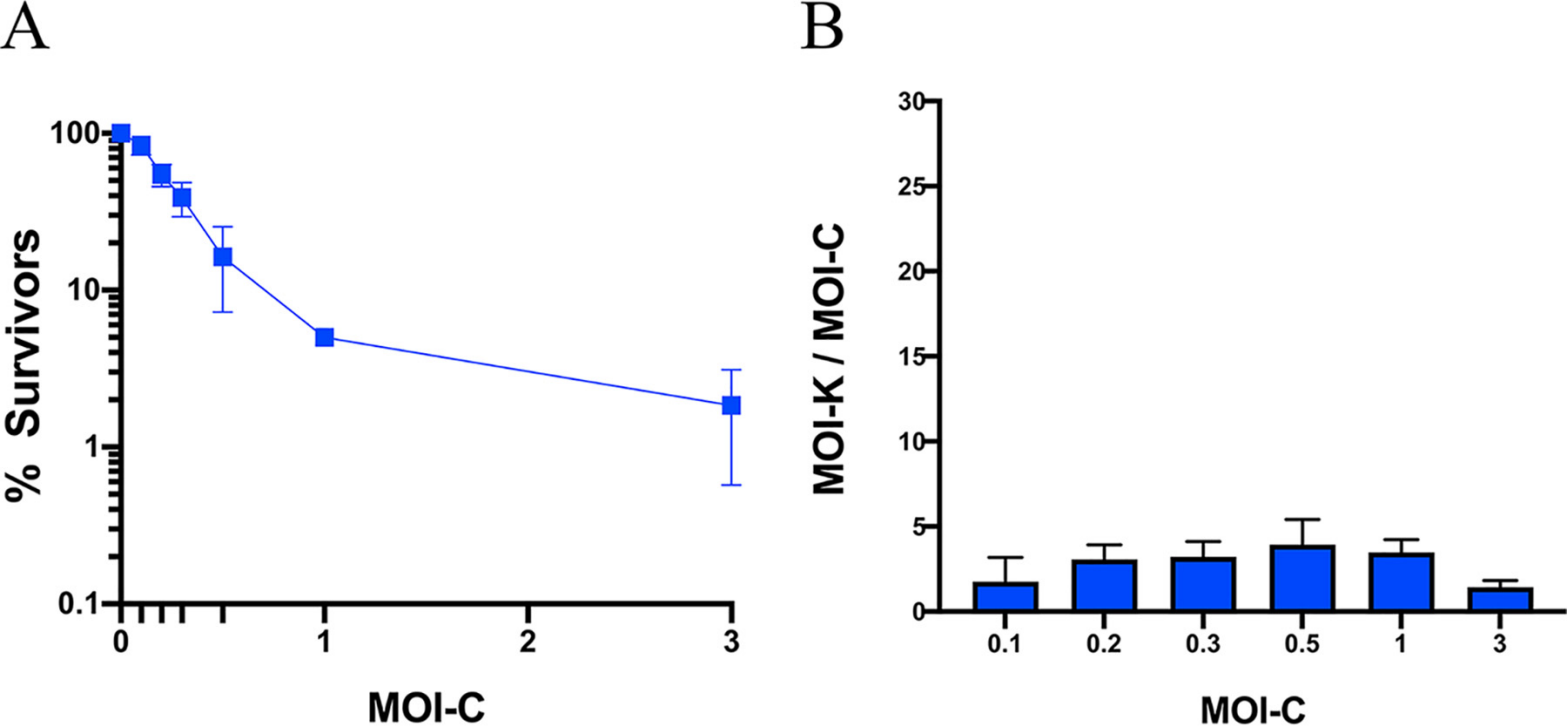
Killing versus transduction by ABD2016. (A) Dose-response curve for killing of RN3 by ABD2016 (*cadA*-tagged ABD2003). RN3 cells in phage buffer were infected with ABD2016 (Cd^r^) at different MOI-C. Uninfected cells (survivors) were enumerated by plating on TSB. ND57, an isogenic *Δagr* mutant, was used to determine ABD2003 transduction titers. Graph represents data from three independent experiments. (B) Graph represent ratios of killing MOI (MOI-K) versus MOI calculated using Cd^r^. MOI-K were calculated by the Poisson formula for surviving fractions from panel A.

Accordingly, we exchanged the ABD selective marker from *tetM* to *cadA* (13) and found that the transduction frequency with CdCl_2_ selection did not increase during preincubation in nonselective medium, was equivalent to that obtained with maximum *tetM* expression (Fig. 5), and was not multiplicity dependent (Fig. 5B). Moreover, the remarkable establishment pattern seen with ABD2031 (Tc^r^) was not seen with ABD2034 (Cd^r^), as shown in Fig. 4B and D, respectively. In most wells, establishment occurred before the first generation, and in a few, it appeared to occur one or two generations later. Such short delays would be expected for entry of a single, immediately integrating ABD molecule into a postreplicative cell or into a clump of 2 or 3 cells. There was, however, as with Tc^r^, a similar proportion of wells lacking any transductants, corresponding to the slightly lower frequency of transduction than of killing, as also shown in Fig. 4.

### TetM acts in *trans* to interfere with ABD establishment.

To determine whether this pattern of delayed establishment was a property of TetM, we repeated the fluorescence-activated cell sorting (FACS) experiment with a derivative of ABD2031 in which *tetM* was replaced by *cadA*. As shown in Fig. 4B, the microtiter wells in which single green cells had been deposited had either 50 to 100% of green Cd^r^ cells or none, with the latter representing the same fraction of wells that had no transductants in the initial experiment with *tetM*, suggesting that a *cadA*-containing ABD established immediately following entry or never. This result suggested that the *tetM* gene, per se, was responsible for the remarkable pattern of ABD establishment shown in Fig. 4A, raising the question of whether the gene acted in *cis* or its product acted in *trans* to interfere with ABD establishment. This was addressed in an experiment in which establishment of a *cadA-*labeled ABD was tested in a strain with *tetM* in *trans* versus a strain lacking *tetM.* As shown in Fig. 4E and F, delayed establishment in a majority of wells was seen with *tetM* in *trans*, similar to that seen with *tetM* in *cis* (Fig. 4A and B), whereas in the absence of *tetM*, ABD establishment was immediate or never, as shown above. This result suggests that the *tetM* gene somehow interferes with ABD establishment, possibly via the gene product, TetM. We note that TetM interferes with Tc inhibition by modifying ribosome structure to dislodge Tc (15, 16), a mechanism that does not immediately suggest how it might interfere with ABD establishment.

### Abortive transduction with a plasmid-carried protospacer.

**FIG 6:**
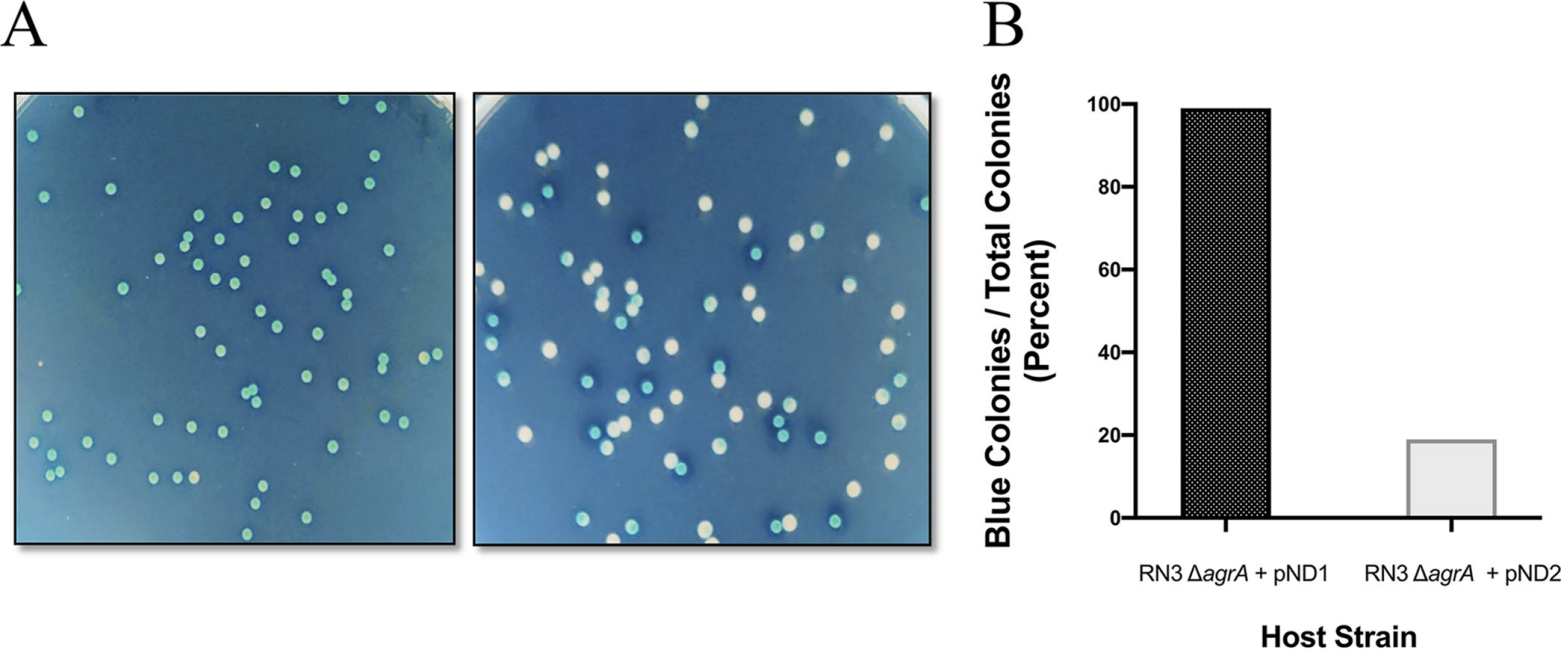
Blue-white colony screening of ABD-infected cells. (A) RN3 Δ*agrA*+pND1 (ND58; left) and RN3 Δ*agrA*+pND2 (ND59; right) were infected with ABD2016 (*cadA*-tagged ABD2003) (Cd^r^) at an MOI-C of 2. The transduction titers were calculated in RN3 Δ*agrA* mutant on GL-Cd100 selection plates. The infection tubes were incubated at room temperature for 30 min, followed by serial dilution and plating of 100 μl of 10^−5^ dilution on TSB X-Gal. The plates were incubated at 37°C overnight. (B) The percentage of blue cells versus total cells was calculated and correlated with survivors on one-hit killing curve assay.

To confirm the observation that some 50% of entering ABD DNA molecules express their cargo gene(s) but fail to generate stable transductants, we constructed a plasmid that contained the *agrA* protospacer, so that in a Δ*agr* host cell, this plasmid would be eliminated by the CRISPR/*cas9/agrA* module of ABD2003 without harming the cell (Fig. S4). To enable blue/white colony scoring, the plasmid encoded β-galactosidase, and to improve plasmid stability, it contained the theta replicon of pI258 (13). This plasmid, pND2, which has a copy number of about 5, was transferred to ND57 (RN3 Δ*agr*), and the resulting strain tested by infection with ABD2016 (*cadA*-tagged ABD2003) at an MOI-C of 2, with which 85% of cells would be infected. ABD-infected cells were plated on TSB 5-bromo-4-chloro-3-indolyl-β-d-galactopyranoside (X-Gal) plates, both with and without CdCl_2_. On an X-Gal plate without Cd there were 3 types of colonies; ∼15% were fully blue, representing uninfected cells, and ∼50% were fully white, representing ABD-infected cells in which the *agrA* protospacer of the plasmid was cleaved immediately in all five copies of the plasmid, eliminating the plasmid. The remaining 35% were white with blue sectors, representing ABD infection and plasmid cleavage in cells that were either postreplicative or belonged to a small clump. Replica plating revealed that about half of the white or sectored colonies were totally Cd^s^. These had lost the plasmid, owing to Cas9 cleavage, but had not retained the ABD, confirming that the ABD could enter a cell, express its lethal cargo, and then disappear, failing to generate a transductant. One possible mechanism for the establishment failure is inefficient circularization of the incoming ABD DNA. This possibility is consistent with the results of an experiment with a *recA-*deficient mutant of an NCTC8325 derivative. With this mutant the transduction frequency was reduced 5-fold, but the killing efficiency was unaffected (Fig. S5). Experiments are currently in progress to test directly for circularization. The remaining 35% of the colonies were Cd^r^, having lost the plasmid owing to Cas9 cleavage and retained the ABD as an integrant. The entirely blue colonies on X-Gal plates lacking CdCl_2_, about 15%, was very close to the percentage of uninfected cells expected from the Poisson distribution (Fig. 6, Fig. S6). With a control plasmid lacking the *agrA* protospacer, the blue/white ratio was about 99:1, with 1% of white colonies representing spontaneous plasmid loss.

10.1128/mBio.02083-21.4 FIG S4 Plasmid map of pND1 and pND2. Download FIG S4, PDF file, 0.1 MB. Copyright © 2021 Dhasmana et al. 2021 Dhasmana et al. https://creativecommons.org/licenses/by/4.0/ This content is distributed under the terms of the Creative Commons Attribution 4.0 International license.

10.1128/mBio.02083-21.5 FIG S5 Effect of RecA on ABD transduction and killing efficiencies. (A) Transduction titer was confirmed by mixing equal numbers of ABD2001 particles with RN450 and RN450 Δ*recA* strains for 30 min and plating on TSB Tc5. Graphs represent data from two independent experiments. (B) Equal numbers of ABD2003 particles were mixed with 100 μl of cells, and survivors were calculated using CFU numbers on TSB agar plates. Download FIG S5, PDF file, 0.1 MB. Copyright © 2021 Dhasmana et al. 2021 Dhasmana et al. https://creativecommons.org/licenses/by/4.0/ This content is distributed under the terms of the Creative Commons Attribution 4.0 International license.

10.1128/mBio.02083-21.6 FIG S6 Blue-white colony screening of ABD-infected cells. The RN3 Δ*agrA*+pND1 (ND58; left) and RN3 Δ*agrA*+pND2 (ND59; right) were infected with ABD2016 (*cadA*-tagged ABD2003) (Cd^r^) at an MOI-C of 0.3. The transduction titers were calculated in the RN3 Δ*agrA* strain on GL-Cd100 selection plates. The infection tubes were incubated at room temperature for 30 min, followed by serial dilution and plating of 100 μl of 10^−5^ dilution on TSB X-Gal. The plates were incubated at 37°C overnight. (B) The percentage of blue cells versus total cells were calculated and correlated with survivors on one-hit killing curve assay. Download FIG S6, PDF file, 0.7 MB. Copyright © 2021 Dhasmana et al. 2021 Dhasmana et al. https://creativecommons.org/licenses/by/4.0/ This content is distributed under the terms of the Creative Commons Attribution 4.0 International license.

### ABD establishment in native clinical strains.

The inherent and irreducible discrepancy between transduction and killing has turned out to represent a very significant feature of SaPI/ABD biology. With NCTC8325, our standard lab strain, and its derivatives, this difference was around 2-fold; with several clinical strains it was much greater, as shown below.

**FIG 7:**
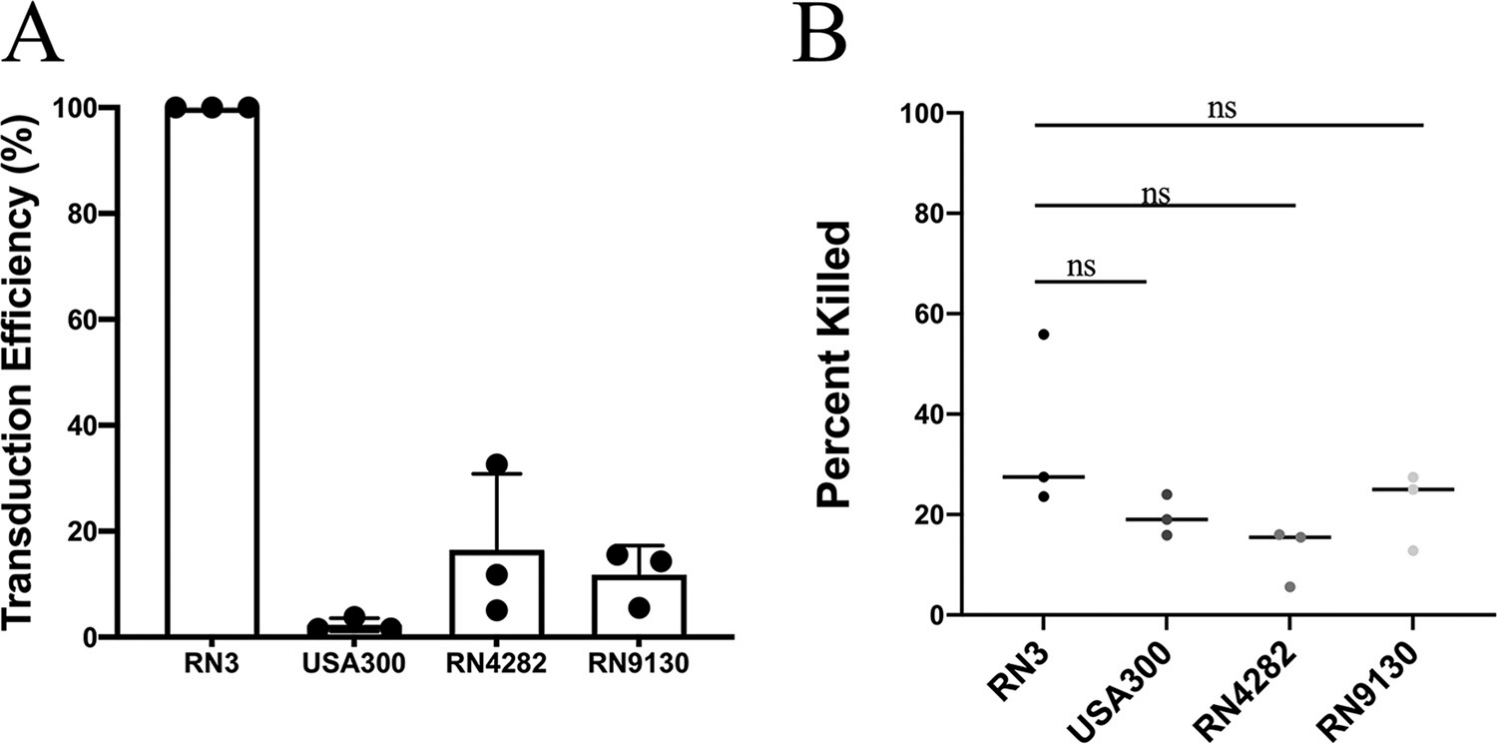
ABD establishment in native clinical strains. (A) The transduction titers were calculated using ABD2016 (*cadA*-tagged ABD2003) (Cd^r^) particles in RN3 Δ*agrA*, USA300 Δ*agrA*, RN4282 Δ*agrA*, and RN9130 Δ*agrA* strains. The graph represents data from three independent experiments. (B) The killing assays were performed using the same number of particles (RN3 titers for an MOI-C of 1). Two-tailed paired *t* test was used. Significant *P* value was set at <0.5.

Our studies with the ABD system were begun with our standard lab strains, mostly derivatives of NCTC8325 that are killed by ABD2003 (CRISPR/*cas9/agrA*) with essentially 100% efficiency. Among clinically important native Staphylococcus aureus strains, only about 1/3 show this level of sensitivity. For the others, the frequency of ABD killing was 5 or 6 orders of magnitude lower, largely owing to ABD DNA destruction by restriction enzymes. A separate study addressing restriction incompatibility is in progress. (There is also a third set, CC395 strains [17], also not studied here, which do not adsorb 80α or the ABD particles.) Among the strains that were 100% sensitive to ABD killing, transduction efficiency was quite variable, ranging from ∼3% of killing efficiency to the ∼50% seen with NCTC8325 strains, as described above. Two exemplary strains are Newman and USA300, which show ∼30-fold lower frequency of ABD transduction than of Cas9 killing (Fig. 7). Although the differences in killing efficiencies are not significant, they are below the predicted killing from Poisson’s distribution, which could be because of cellular clumping. A sorting experiment similar to that shown in Fig. 4 revealed that only 1 of 24 single green cells deposited in microtiter plate wells gave rise to a culture containing detectable green cells, suggesting that the fraction of ABDs failing to establish stable transductants in these strains was over 95% and that abortive transduction was simply that much more likely. To rule out the possibility that the negative wells were due to accidental plating of non-ABD-containing cells, we used plasmid ND2 as a target for CRISPR/*cas9/agrA* in an *agr-*negative host (USA300 Δ*agr*::*tetM*) and found two classes of ABD2016 transductants on an X-Gal-TSB plate, USA300, those that were white but remained Cd^s^ and those that were white and were Cd^r^. As in the above-described experiment, the white colonies contained various proportions of blue cells, again owing to the existence of small clumps in the USA300 culture. The white Cd^r^ colonies represent cells in which the plasmid had been eliminated by Cas9 cleavage and the ABD had integrated into its *att* site, and the white CdS colonies represent those in which the plasmid had been eliminated, owing to rapid expression of *cas9*, but ABD integration had failed. These numbers closely matched the transduction results.

### What might be responsible for blocking ABD DNA establishment?

The dramatic interstrain difference in blocking suggested that one or more specific genes were responsible, which could be chromosomal, SaPI, plasmid, or prophage genes. The identification and characterization of these putative ABD-blocking genes is the subject of a separate investigation; preliminary evidence with strain Newman suggests that in at least one case, a prophage was responsible.

**FIG 8:**
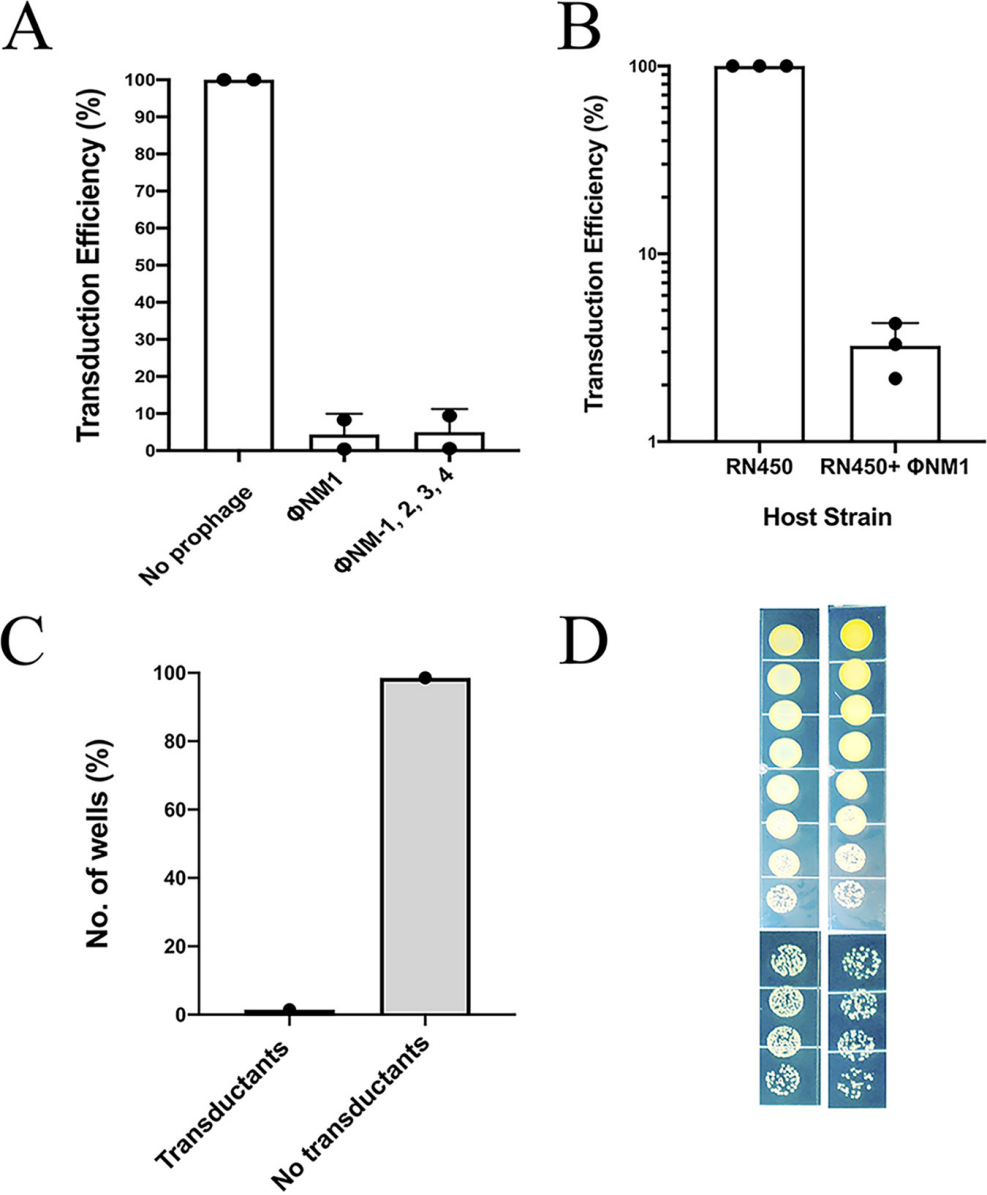
Prophage NM1-mediated interference in ABD establishment in S. aureus Newman strain. (A) Transduction titers of ABD2015 (Cd^r^) calculated in Newman cured of all prophages, Newman with ϕNM1 only, and Newman with all four prophages, ϕNM1-4. The bar graph represents data from three independent experiments. (B) Transduction titers of ABD2015 (Cd^r^) calculated in RN450 and RN450 lysogenized with prophage NM1. (C) The graph represents wells with transductants and no transductants upon single-cell sorting of GFP-labeled ABD2031 (Tc^r^)-infected Newman NM1 strain. The spot tests were performed on both TSB plain and TSB Tc5 plates. (D) Dilution plating of H9 wells showing total number of cells versus transductants.

Strain Newman is a much-studied ABD-sensitive clinical strain that harbors 4 different prophages, one of which, ϕNM1, had been observed to interfere with SaPI transfer (18). We had previously compared a Newman derivative cured of all 4 prophages (5) with the parental strain and found that the phage-related interference applied to the ABDs as well (Fig. 8). We prepared an ϕNM1 lysogen of RN450 and found that it reduced transduction of ABD2015 by about 30-fold (Fig. 8) but, like the other ABD-sensitive clinical strains, had no effect on the bactericidal activity of ABD2016 (Fig. S7). We performed a cell-sorting and plating test with RN12410 (ϕNM1) and found that only one of the 70 microtiter wells containing bacteria had any ABD transductants, consistent with the 30-fold reduction in transduction titer seen with this strain. Remarkably, this single well (H9) contained >60% ABD^+^ cells (Fig. 8), indicating that the integration event had taken place before the first cell division and that ABD establishment had failed in all of the other wells. This result indicates that in at least one strain, a prophage can block ABD establishment. Prophages and other MGEs in other strains showing this phenomenon are currently under study.

10.1128/mBio.02083-21.7 FIG S7 Comparison of ABD killing efficiencies in RN450 and RN450 ɸNM1. Equal numbers of ABD particles were mixed with 100 μl of cells, and survivors were calculated using CFU numbers on TSB agar plates. Download FIG S7, PDF file, 0.08 MB. Copyright © 2021 Dhasmana et al. 2021 Dhasmana et al. https://creativecommons.org/licenses/by/4.0/ This content is distributed under the terms of the Creative Commons Attribution 4.0 International license.

## DISCUSSION

The mechanisms of horizontal gene transfer in bacteria were worked out many years ago, and one might have thought that there was little or nothing more to be learned. However, a close look at one particular example, phage-mediated transfer of staphylococcal pathogenicity islands and their ABD derivatives, has revealed several unexpected and important novel features of this process. Indeed, in this study, we report that this presumably simple process is actually a complex, multifaceted one, bound up with the selective marker used for scoring transfer and the fate of the transferred DNA. To summarize, we began with *tetM* as the selective marker and found that it significantly underestimates transfer frequency. One problem is that as *tetM* is ordinarily used, insufficient time is provided for the full expression of resistance before challenge with Tc, owing to Tc inhibition of protein synthesis. This was easy enough to remedy, either by providing the *tetM* inducer, anhydrotetracycline, or allowing more time for Tc^r^ expression or by substituting *cadA* for *tetM*. More importantly, we discovered that the *tetM* gene interferes with the establishment (integration?) of a transferred genetic element, causing it to reside in the recipient cell for as many as 30 generations before integrating into its chromosomal *att* site. This interference turned out to be unrelated to Tc^r^, as it was seen in the absence of the drug in an experiment using FACS to purify cells newly receiving a *gfp*-labeled ABD, depositing single green cells in microtiter wells where they were allowed to grow in the absence of selection. After overnight growth, each well contained ∼10^9^ cells, of which a highly variable fraction contained the ABD. This remarkable pattern of stochastic establishment was not seen when *tetM* was replaced by *cadA*, which enabled immediate establishment of an incoming ABD. The interference by *tetM* could have been a *cis* effect, such as interfering with local transcription, or it could have been a *trans* effect of the *tetM* gene. The latter turned out to be the case, by a far from obvious mechanism, given that TetM is a highly sequence-specific RNA-binding protein.

A third surprising finding was the marker-independent failure of a significant fraction of incoming ABD DNA molecules to establish stable transductants. The frequency of this was highly strain dependent, ranging between 50 and 97% in the few strains examined, and, in one case, was greatly increased by a prophage. Though destined to fail, these incoming molecules immediately expressed their cargo genes, including lethal *cas9* or a reporter such as GFP. To confirm that these failure-bound ABD molecules could express their cargo genes, we used a plasmid containing a protospacer as a Cas9 target and found that the plasmid was eliminated by Cas9, despite the establishment failure of the ABD molecule carrying it. In retrospect, this is reminiscent of the well-characterized phenomenon of abortive transduction, in which incoming chromosomal fragments express their genes and are inherited linearly for at least several hours but never recombine with their chromosomal homologs (19). In classical abortive transductants, obtained with phage P1-mediated transduction, the abortive fragment is circular and supercoiled, with its ends connected by a protein (20), a configuration that would protect the DNA from exonuclease degradation, would probably inhibit recombination, and is presumably responsible for prolonged linear inheritance. Abortive transduction has not, to our knowledge, been reported for mobile genetic elements, and experiments are in progress to determine the structure of the putative abortive ABD transducing fragments. If ABD abortive transductants have prolonged linear inheritance, an interesting therapeutic scenario is suggested in which the ABD integrase would be deleted so that all of the incoming ABD molecules would be abortive; the cargo would be expressed and functional but the element would not be transferred secondarily, would not mobilize any mobile elements in the target organism, and would be inherited linearly. This would be useful only for a lethal cargo that would kill before the first cell division so that there would be no escapees.

In summary, we have encountered several different phenomena in studying the efficiency of transduction versus killing for the ABD. (i) We found a deficiency in transduction frequency for Tc^r^ compared to Cd^r^ owing to inhibition of protein synthesis by Tc but not by Cd. (ii) We also saw very slow and stochastic establishment of a *tetM*-carrying ABD that was not seen with the same ABD carrying *cadA* in place of *tetM*. Since the *tetM*-associated stochastic establishment occurred in the absence of selection during growth following ABD DNA entry, it is not related to tetracycline resistance and is provisionally attributed to the *tetM* gene or its product, per se. (iii) An irreducible, strain-specific, but marker-independent ABD establishment failure, similar to abortive transduction, was seen. We suspect that any incoming DNA that requires integration will show the same establishment failure and that elements not requiring integration, such as lytic phages and plasmids, will not. (iv) In some strains, establishment failure is much more frequent (>95%), in at least one case is attributed to a prophage, and, thus, it resembles other phage-specific types of interference with mobile genetic element (MGE) transfer (21).

It appears that these phenomena are independent of initial gene expression by the ABD DNA following entry and will not impact its bactericidal utility. They may, however, adversely impact the ability of an incoming ABD molecule to disable its target organism, block biofilm formation, etc.; an obvious possible remedy is to equip the ABD with an addiction module (toxin-antitoxin). This is currently in progress.

## MATERIALS AND METHODS

### Bacterial strains and growth conditions.

E. coli strains were grown in Luria-Bertani (9) broth and LB agar plates, while S. aureus strains were grown in CY broth (22) supplemented with 1% glucose, tryptic soy broth (TSB; Difco), TSB agar (Difco), and GL agar (22). Antibiotic-resistant S. aureus cells were selected on 2.5 μg/ml erythromycin (Em2.5), 5 μg/ml tetracycline (Tc5), and 100 μM/200 μM CdCl_2_ (Cd100/Cd200). Antibiotic-resistant E. coli cells were selected on ampicillin at 100 μg/ml (Ap100). The bacterial strains, plasmids, and primers used in this study are listed in Tables 1, 2, and 3, respectively.

**TABLE 1:** Bacterial strains

Strain	Description	Reference
E. coli DH5α	F^−^ *endA1 glnV44 thi-1 recA1 relA1 gyrA96 deoR nupG* Φ80d*lacZ*ΔM15 Δ(*lacZYA-argF*)*U169*, *hsdR17* (r_K_^−^ m_K_^+^), λ^−^	27
RN1	NCTC8325; lysogenic for ϕ11, ϕ12, and ϕ13	13
RN3	NCTC8325; lysogenic for ϕ12 and ϕ13	Lab collection
ND57	RN3 Δ*agr*::*tetM*; lysogenic for ϕ12 and ϕ13	This study
ND58	ND57 (pND1)	This study
ND50	ND57 (pND2)	This study
RN11	RN1 (pI258); lysogenic for ϕ11, ϕ12, and ϕ13	13
RN450	NCTC8325; cured of ϕ11, ϕ12, and ϕ13	28
RN6911	RN450 Δ*agr*::*tetM*	Lab collection
RN4220	Restriction-defective derivative of RN450	29
RN12064	RN450 80α Δ*terS* Δ*agr*::*cadA*	5
RN12065	RN12064 (ABD2002)	5
RN12066	RN12064 (ABD2003)	5
RN12156	RN450 (80α Δ*terS*) (ABD2001)	5
RN12407	RN12064 (ABD2030)	This study
RN12408	RN12064 (ABD2031)	This study
RN12414	RN3 Δ*agr*::*cadA*	This study
ND78	RN3 (pCN36)	This study
RN12344	RN450 Δ*agr* (80α Δ*terS*)	This study
RN12345	RN12344 (ABD2002)	This study
RN12346	RN12344 (ABD2003)	This study
RN12362	RN12344 (ABD2015)	This study
RN12363	RN12344 (ABD2016)	This study
RN12147	USA300; cured of large plasmid	30
ND63	RN12147 Δ*agr*::*tetM*	This study
RN4282	Clinical TSS isolate; contains SaPI1	Lab collection
RN7470	RN4282 Δ*agr*::*tetM*	Lab collection
RN9130	Nasal carriage isolate 502A	Lab collection
RN9120	RN9130 Δ*agr*::*tetM*	Lab collection
RN10950	S. aureus Newman; cured of ϕNM1, ϕNM2, ϕNM3, ϕNM4	31
RN10951	S. aureus Newman (ϕNM1, ϕNM2, ϕNM3, ϕNM4)	31
RN12409	S. aureus Newman; cured of ϕNM2, ϕNM3, ϕNM4	This study
RN12410	RN450 (ϕNM1)	This study
ND64	RN10950 Δ*agr*::*tetM*	This study
ND65	RN10951 Δ*agr*::*tetM*	This study
ND32	RN450 Δ*recA*	This study

**TABLE 2:** Plasmids and ABDs

Plasmid or ABD serial no.	Description	Reference or source
Plasmid		
pI258	Low-copy-no. plasmid with theta replicon	32
pCN36	E. coli-S. aureus shuttle vector containing *tetM*	25
pCN51	Shuttle vector of E. coli and S. aureus	25
pMAD	Vector for allelic replacement in S. aureus	23
pND1	pCN51 with insertion of *bgaB* with *lac* promoter into BamHI-EcoRI sites, plus replacement of *repC* with pI258 theta replicon	This study
pND2	pND1 Δp*cadA*::*agrA*	This study
pND11	pMAD with flanked *cadA* marker for ABD2001, used for allelic exchange	This study
pND12	pMAD with flanked *gfp* marker for ABD2001, used for allelic exchange	This study
pKM1	pMAD with flanked *cadA* marker for ABD2002, used for allelic exchange	This study
pKM3	pMAD with flanked *cadA* marker for ABD2003, used for allelic exchange	This study
pKM7	pMAD with flanks for *agr* deletion, used for allelic exchange	This study
pND3	pMAD with flanks for *recA* deletion, used for allelic exchange	This study
Serial no.		
ABD2001	SaPI2 Δ*tst::tetM* Δ*ptiB* Δ*cpmAB* Δ*eta*	5
ABD2002	ABD2001::CRISPR/cas9/nts^*a*^	5
ABD2003	ABD2001::CRISPR/cas9/*agrA*	5
ABD2015	ABD2002 Δ*tetM*::*cadA*	This study
ABD2016	ABD2003 Δ*tetM*::*cadA*	This study
ABD2030	ABD2001 Δ*tetM*::*cadA*	This study
ABD2031	ABD2001::*gfp*	This study
ABD2034	ABD2030::*gfp*	This study

a nts, nontargeting spacer.

**TABLE 3:** Primers

Primer and function	Sequence (5′–3′)	Reference
Construction of ABD2031 (GFP-labeled ABD2001)		
UP_2001_fwd	ATGGGGAAGGCCATCCAGCCTCGCGTCGGGCGATATCGCGGATGCTTACTCCTATTGTAC	This study
UP_2001_rev	GAGTAGAATAGAAGTATCAAAAAAAGTATATTTGCAATGATAGATATGATTATCC	This study
TT_fwd	ATCATATCTATCATTGCAAATATACTTTTTTTGATACTTCTATTCTACTCTG	This study
TT_rev	ATAAAAGTTATAAAATAATCTTGTTGGATTAAGAGATTAATTTCCCTAAAAATG	This study
Pro-GFP-BlazTT_fwd	ATCATATCTATCATTGCAAATATACTTTTGCCTCCTAAAATAAAAAGTTTAAATTAAATC	This study
Pro-GFP-BlazTT_rev	TTATCACACACATAAATCTCTATAATGTCACTTTGCTTGATATATGAG	This study
Down_2001_fwd	TTCTCATATATCAAGCAAAGTGACATTATAGAGATTTATGTGTGTGATAATTG	This study
Down_2001_rev	ATCGATGCATGCCATGGTACCCGGGAGCTCGCCATTAATATCTCATTTCTATTTATCAGC	This study
Construction of ABD2030 (*cadA*-tagged ABD2001)		
Up-2001-cad_fwd	GGCCATCCAGCCTCGCGTCGGGCGATATCG*GATCC*GTTGATAGATGATGAAATAAATAC	This study
Up-2001-cad_rev	TTAAAAAATACACTTGAATAAGTGCTTTACCACTTTTTCTGTAATAATTATTAATAAAG	This study
pro-cad-TT_fwd	TAATTATTACAGAAAAAGTGGTAAAGCACTTATTCAAGTGTATTTTTTAATAAATTATTT	This study
pro-cad-TT_rev	ATAAATCTCTATAAGTATATTTGCACCGCAGCTGCTGTAAGTATC	This study
Down-2001-cad_fwd	GCAACGATACTTACAGCAGCTGCGGTGCAAATATACTTATAGAGATTTATGTG	This study
Down-2001-cad_rev	TCGATGCATGCCATGGTACCCGGGAGCTCG*AATTC*CCATTAATATCTCATTTCTATTTAT	This study
Construction of *recA* mutant in RN450		
RecA Up_fwd	GGCCATCCAGCCTCGCGTCGGGCGATATC**GGATCC**TAACAAATCATCATGGCATGG	This study
RecA Up_rev	GTACTATTCTTCGTCACCGAAAGATTTCTCCATATTTTTAATTAC	This study
RecA Down_fwd	GAGAAATCTTTCGGTGACGAAGAATAGTACACAAATTTATATC	This study
RecA Down _rev	TCGATGCATGCCATGGTACCCGGGAGCTC**GAATTC**ACTGATACCGTTGATTCACTTG	This study
Construction of pND1 and pND2 (blue/white screening)		
agrA SphI fwd	CCCCGCATGCATGGAAATTGCCCTCGC	This study
agrA SalI rev	CCCCGTCGACTTATATTTTTTTAACGTTTCTCACCGATG	This study
P-BgaB BamHI fwd	CCCCGGATCCCGATAGATCTGTCTAGTTAATGTGTAAC	This study
P-BgaB EcoRI rev	CCCCGAATTCCTAAACCTTCCCGGCTTC	This study
RepO-pI258 Nar1 fwd	CCCCGGCGCCTAGTTTACATAATCGATGATTACCAGAAG	This study
RepO-pI258 ApaI rev	CCCCGGGCCCCTTCTTCGTCTGTCGTTTTATC	This study
Construction of ABD2015 (*cadA*-tagged ABD2002)		
5′pMAD_agrUP 2	AGGCCATCCAGCCTCGCGTCGGGCGATATCGGATCCGACTGTAGATTTAAACTTAAATGA	This study
3′agrUP 2	AATACGCCGTTAACTGACTTTATTATCTTATATATTGCCTAACTGTAGGAAAT	This study
5′agrDN 2	AGTATTTATTTCCTACAGTTAGGCAATATATAAGATAATAAAGTCAGTTAACGG	This study
3′pMAD_agrDN	ATCGATGCATGCCATGGTACCCGGGAGCTCGAATTCCTCTGCTGATATGTTATTTGA	This study
5’pMAD_ABDUp	GGCCATCCAGCCTCGCGTCGGGCGATATCGGATCCGTTGATAGATGATGAAATAAATACC	This study
3′_CadA_ABDUp	TTAAAAAATACACTTGAATAAGTGCTTTACCACTTTTTCTGTAATAAT	This study
Construction of ABD2016 (*cadA*-tagged ABD2003)		
5’ABD_CadA	ATTAATAATTATTACAGAAAAAGTGGTAAAGCACTTATTCAAGTGTATTTTTTA	This study
3′ABD_CadA	AGTAATATTGACTTTTAAAAAAGGCTCGAGCCGCAGCTGCTGTAAGTAT	This study
5’CadA_ABD_DN	TGATCGCAACGATACTTACAGCAGCTGCGGCTCGAGCCTTTTTTAAAAGTCAA	This study
3’pMAD_ABD_DN	TATCGATGCATGCCATGGTACCCGGGAGCTCGAATTCTAAAATCCGTTAAAGAGTTACT	This study
Construction of ABD2034 (*gfp*-tagged ABD2030)		
UP 2001-C_fwd	TAATGGGGAAGGCCATCCAGCCTCGCGTCGGGCGATATCGGATCCCTATCATTACAGCTTTAGAATATCGTTC	This study
UP 2001-C_rev	GAATAGAAGTATCAAAAAAAGTATATTTGCACCGCAGCTG	This study
GFP_fwd	CAGCTGCGGTGCAAATATACTTTTTTTGATACTTCTATTCTACTCTG	This study
GFP_rev	ACACACATAAATCTCTATAATGTCACTTTGCTTGATATATGAG	This study
Dn 2001-C_fwd	ATATATCAAGCAAAGTGACATTATAGAGATTTATGTGTGTGATAATTG	This study
Dn 2001-C_rev	CACATTAACTAGACAGATCTATCGATGCATGCCATGGTACCCGGGAAACTATCTATTGTTACCATTAATATCTC	This study

### Preparation of ABD lysates.

Particle lysates were prepared by growing the strains to mid-log phase, optical density at 600 nm (OD_600_) of 0.6, and diluted to an OD_600_ of 0.3 with phage buffer. ABD-containing lysogens were induced with 1.5 μg/ml mitomycin C (MC) (Sigma); 80α nonlysogens were lysed by phage 80α at an MOI of 0.1. Cultures were incubated at 32°C with slow shaking until complete lysis (usually 3 to 4 h). The lysates were then treated with DNase for 4 h at 37°C and filter sterilized using a 0.22-μm syringe filter. The ABD particles were concentrated with polyethylene glycol 8000 (PEG8000) and purified by CsCl density step gradient centrifugation.

### Density gradient purification of ABD particles.

ABD particles were prepared by MC induction of ABD-containing RN12064 derivatives. Lysates were treated with DNase I (50 μg/liter) for 4 h at 37°C; particles were precipitated overnight at 4°C using 8% PEG8000 and 0.4 M NaCl (final concentrations). The precipitated particles were centrifuged at 10,000 rpm for 10 min at 4°C in a Sorvall RC2B centrifuge. The pellets were resuspended in phage buffer and purified by centrifugation at 30,000 rpm for 3 h (rotor-TH641) through a CsCl step gradient formed with 1 ml of 1.6 g/ml, 2 ml of 1.5 g/ml, 2 ml of 1.4 g/ml, and 3 ml of 1.3 g/ml CsCl. Particles were dialyzed in two steps. The first dialysis was carried out against phosphate-buffered saline (PBS) supplemented with 1 M NaCl overnight, while the second dialysis was for 1 h against PBS only. An equal volume of phage buffer was added to increase shelf life, and the particles were stored at 4°C thereafter. Particle titers were determined by transduction with selection for tetracycline (Tc^r^) or cadmium resistance (Cd^r^).

### Transduction.

Transductions were performed as previously described (5). Briefly, recipient cells were resuspended in phage buffer at an OD_600_ of 2; 100 μl of cells was infected with different dilutions of purified ABD particles and kept at room temperature for 30 min. Thereafter the infected cells were mixed with 3 ml of molten top agar and plated on selective agar plates. Throughout the text, MOI-T refers to multiplicity of infection based on standard transduction with selection for Tc^r^, MOI-C refers to MOI based on transduction with selection for Cd^r^, and MOI-K refers to the killing MOI calculated using survivors from killing curves.

### ABD DNA modification.

**(i) Construction of GFP-labeled ABD DNA.** For deletion of specific genes, the 1-kb left fragment and 1-kb right fragment of selected gene to be deleted were amplified by PCR and cloned in pMAD using Gibson assembly master mix (7). For integration of GFP into ABD, 1-kb left fragment, GFP gene, and 1-kb right fragment were amplified using PCR and were cloned in pMAD in the same order using Gibson assembly master mix (23). The mix was transformed in Top10 chemically competent cells and the colonies were selected on LB plates supplemented with Ap100. The pMAD plasmids obtained were confirmed by sequencing. RN4220 was electroporated with pMAD plasmid and selected on TSB Em2.5 plates. Transformant colonies were streaked on TSB plates containing Em2.5 plus X-Gal 150 μg/ml and incubated at 32°C. Blue colonies containing recombinant plasmids were picked and resuspended in phage buffer at an OD_600_ of 0.3, and the recombinant pMAD plasmids were transduced to RN450 with phage 80α selecting for EmR at 32°C.

Recipient cells were resuspended in phage buffer, 100 μl of phage lysate was added and the mix was incubated at room temperature for 30 min. A volume of 100 μl of 1 M sodium citrate was added and incubated at 32°C with slow shaking for 90 min. A volume of 3 ml top agar was added and poured on TSB Em2.5 plates. The plates were incubated at 32°C overnight. The colonies obtained were streaked on TSB Em2.5 plus 150 μg/ml X-Gal. A blue colony was selected and grown overnight at 32°C in TSB supplemented with Em2.5. A volume of 100 μl of 10^−3^ and 10^−4^ dilutions were plated on to TSB Em2.5 plus X-Gal at 150 μg/ml and incubated at 42°C for first crossover. A light blue colony was picked and grown in TSB and incubated at 32°C overnight. A volume of 100 μl of 10^−5^ and 10^−6^ dilutions were plated on a TSB plate supplemented with 150 μg/ml X-Gal. The plates were incubated at 42°C for second crossover. White colonies were selected and patched on TSB Em2.5 plus X-Gal at 150 μg/ml. The obtained colonies were screened for correct integration or deletion by PCR.

**(ii) Allelic replacement of antibiotic resistance markers.** To generate a clean deletion of the *agr* operon in the particle-producing strain, we PCR-amplified homology regions flanking the *agr* operon using primers 5′pMAD_agrUP 2 and 3′agrUP 2 upstream and 5′agrDN 2 and 3′pMAD_agrDN downstream from RN1. The fragments were gel purified, introduced into plasmid pMAD by Gibson assembly at the BamHI and EcoRI sites, and transformed into E. coli DH5α, resulting in the plasmid pKM7 (7). The plasmid was then electroporated into RN4220. A lysate was prepared from the strain carrying pKM7, filtered, and used to transduce the plasmid into the ABD particle-producing strain (RN12064). Allelic replacement was then performed using a standard protocol (24). Candidate colonies were screened for allelic exchange using PCR amplification and confirmed by sequencing.

To replace the tetracycline resistance marker (*tetM*) with a cadmium resistance marker (*cadA*) on ABD2001, ABD2002, and ABD2003, we PCR amplified upstream and downstream sequences with homology to each ABD using primers 5′_pMAD_ABD-UP and 3′_CadA_ABD-UP upstream and 5′CadA_ABD-DN and 3′pMAD_ABD-DN downstream from strains containing ABD2001, ABD2002, and ABD2003. We then PCR amplified the *cadA* gene and its promoter from RN11 (containing plasmid pI258) using primers 5’ABD_CadA and 3’ABD_CadA. Each fragment was gel purified and introduced into plasmid pMAD by Gibson assembly at the BamHI and EcoRI sites (7). Each was transformed into E. coli DH5α, resulting in a plasmid with homology to ABD2001 as plasmid pND11, ABD2002 as pKM1, and a plasmid with homology to ABD2003 as pKM3. These plasmids were then electroporated into RN4220. Lysates were prepared from the strains carrying pND11, pKM1, and pKM3, filtered, and used to transduce the plasmids into ABD particle-producing strains (RN12156, RN12345, and RN12346, respectively). Allelic replacement was then performed as described above. The resulting ABDs were designated ABD2030, ABD2015, and ABD2016, respectively.

### Construction of plasmid-based assay for blue-white colony screening.

We used pCN51 vector (25) and replaced the rolling-circle replicon with a theta replicon, which was amplified from plasmid pI258 (13). Subsequently, a β-galactosidase gene was amplified from vector pMAD and cloned in BamHI and EcoRI restriction sites. This plasmid was named pND1. Next, we replaced the *cadA* promoter with *agrA* in pND1 and named it pND2. Both pND1 and pND2 were introduced into RN3 Δ*agrA*::*tetM* (ND57) and named ND58 and ND59, respectively. ND58 and ND59 cells were infected with ABD2016 at an MOI-C of 2, followed by incubation at room temperature for 30 min. Serial dilutions were prepared and plated onto TSB X-Gal plates for blue/white colony screening. ABD2016 upon its entry cleaves the *agrA* locus in pND2, causing its degradation, giving white colonies on TSB X-Gal plates. The blue colonies were counted and used to calculate the percentage of noninfected cells.

### Fluorescence microscopy.

RN450 was revived on TSB plates from cryostocks. After overnight incubation at 37°C, cells were suspended in phage buffer at an OD_600_ of 1. RN450 cells were infected with GFP-labeled ABD2001 (Tc^r^) particles at an MOI-T of 0.3. The cell and particle mix was incubated at room temperature for 30 min. The cells were washed with PBS twice and resuspended in PBS. Appropriate dilution was made for the preparation of microscopic slides. The slides were coated with 1% soft agar, and 10 μl of infected cells was applied and covered with coverslips. The cells were visualized under a Zeiss Axio fluorescence microscope under ×63 magnification. To visualize GFP expression in bacterial colonies grown on TSB/GL plates after overnight incubation, a Zeiss Axio Zoom fluorescence microscope was used.

### Single-cell sorting using FACS.

The recipient cells were resuspended in phage buffer under nongrowing conditions. The cells were mixed with GFP-labeled ABD2031 (Tc^r^) or GFP-labeled ABD2034 particles (Cd^r^) at an MOI of 0.3 and incubated at room temperature for 30 min. One milliliter of CYGP broth was added to the infected cells and incubated at 37°C for 30 min for GFP expression. Cells were washed with PBS twice and resuspended in PBS. The appropriate dilution of cells (1.5 × 10^7^/ml) was prepared for FACS. Cells showing GFP signal were sorted using FACS Aria Ilu SORP, and single cells were plated on 96-well plates containing 150 μl of TSB. The seeded plates were incubated at 37°C overnight. Serial dilutions of each well were prepared in phage buffer, and 10 μl of each dilution was spotted onto TSB plain as well as TSB Tc5 plates. The ratios of transductants to total number of cells were calculated using CFU numbers, and percent transductant values were calculated for each well. The values of percent transductants were then used to calculate generations before ABD integrations.

### Statistical analysis.

All statistical analyses were performed in GraphPad Prism 9.2 (26). To test for significance between two groups, two-tailed paired *t* test was used. A significant *P* value was set at <0.5.
