# Supplementary material for: Dynamics of Antibacterial Drone Establishment in Staphylococcus aureus: Unexpected Effects of Antibiotic Resistance Genes
Source: mBio. 2021 Nov 16;12(6):e02083-21. doi: 10.1128/mBio.02083-21 (PMC8593670; doi:10.1128/mBio.02083-21)
Supplement: FIG S1 [file mbio.02083-21-sf001.pdf]

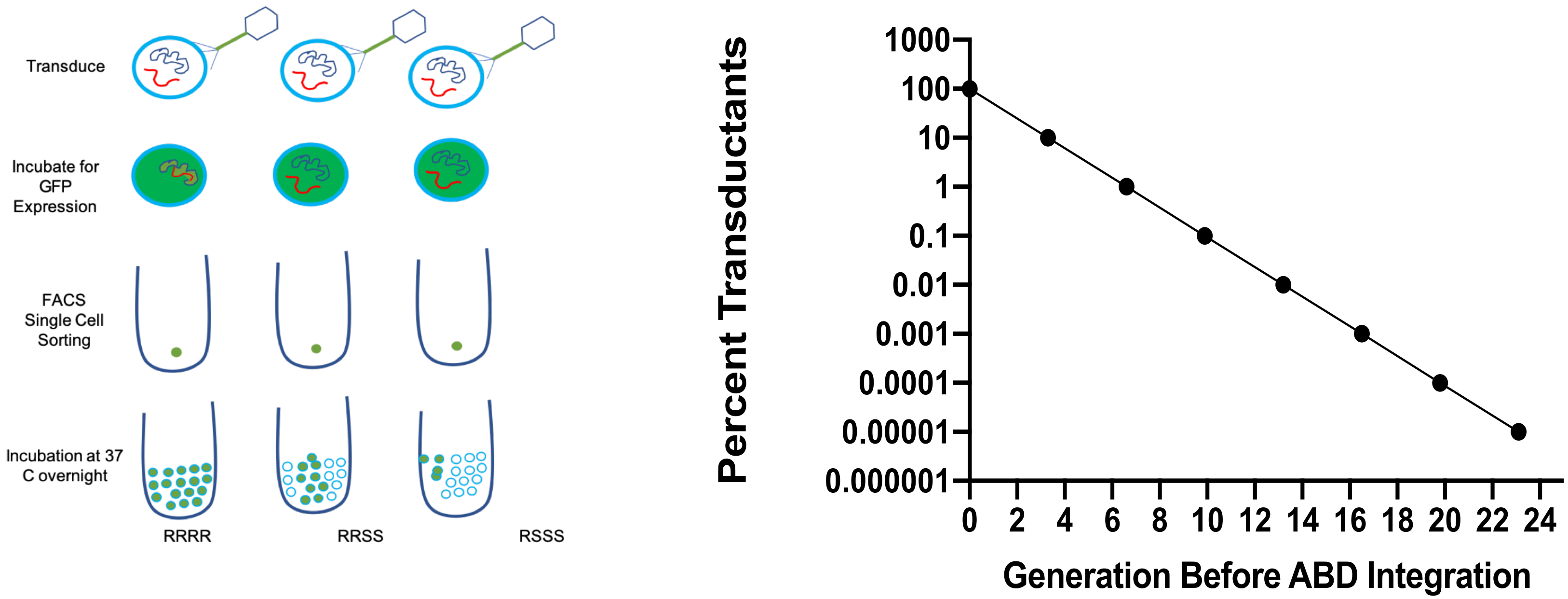


**Figure S1. Strategy used to study ABD integration using single cell sorting with FACS.** NCTC8325 cells were infected with GFP-labeled ABD2031 (TcR) or GFP-labeled ABD2034 (CdR). The infected cells were incubated for 30 minutes for GFP expression and single GFP$^+$ cells were sorted by FACS. These single GFP$^+$ cells were placed in each well of 96-well plate and incubated at 37°C overnight. The generation before ABD integration were calculated using the standard curve.
