# Supplementary material for: Dynamics of Antibacterial Drone Establishment in Staphylococcus aureus: Unexpected Effects of Antibiotic Resistance Genes
Source: mBio. 2021 Nov 16;12(6):e02083-21. doi: 10.1128/mBio.02083-21 (PMC8593670; doi:10.1128/mBio.02083-21)
Supplement: FIG S2 [file mbio.02083-21-sf002.pdf]

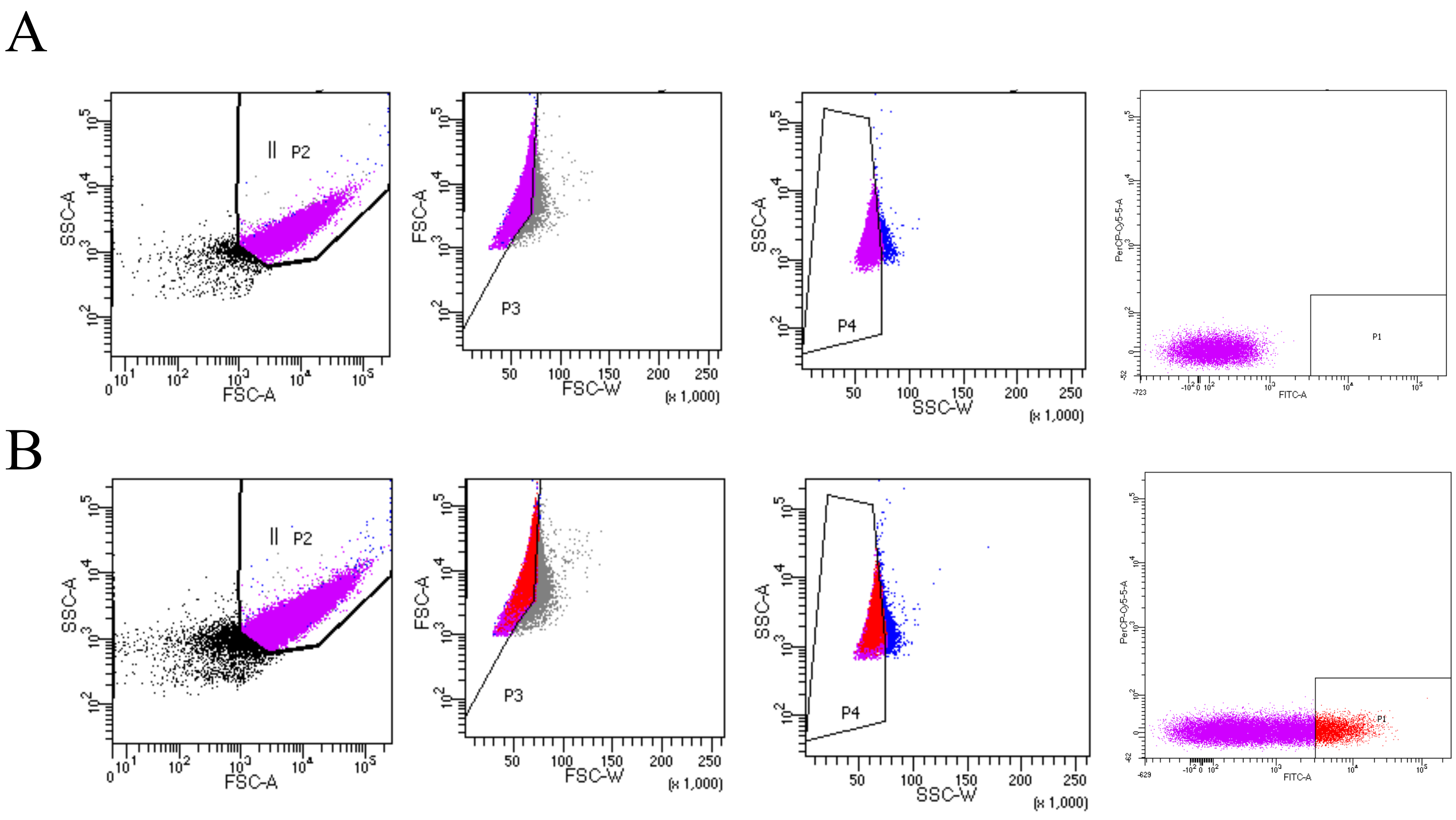

Figure-S2: Gating strategy for isolation of ABD infected cells. RN3 cells infected by ABD2031 or ABD2034 were sorted by FACS, gating on GFP. (**A)** Uninfected cells were used to determine the specific gate (P1) for GFP-positive cells. **(B)** ABD2031 (TcR) or ABD2034 (CdR) infected cells were sorted using a GFP filter in the FACS Aria cell sorter (70mm nozzle). Single GFP-positive cells from the P1 gate were placed in the wells of 96-well microtiter plate containing 150µl of TSB and incubated at 37°C overnight.
