# Supplementary material for: Dynamics of Antibacterial Drone Establishment in Staphylococcus aureus: Unexpected Effects of Antibiotic Resistance Genes
Source: mBio. 2021 Nov 16;12(6):e02083-21. doi: 10.1128/mBio.02083-21 (PMC8593670; doi:10.1128/mBio.02083-21)
Supplement: FIG S3 [file mbio.02083-21-sf003.pdf]

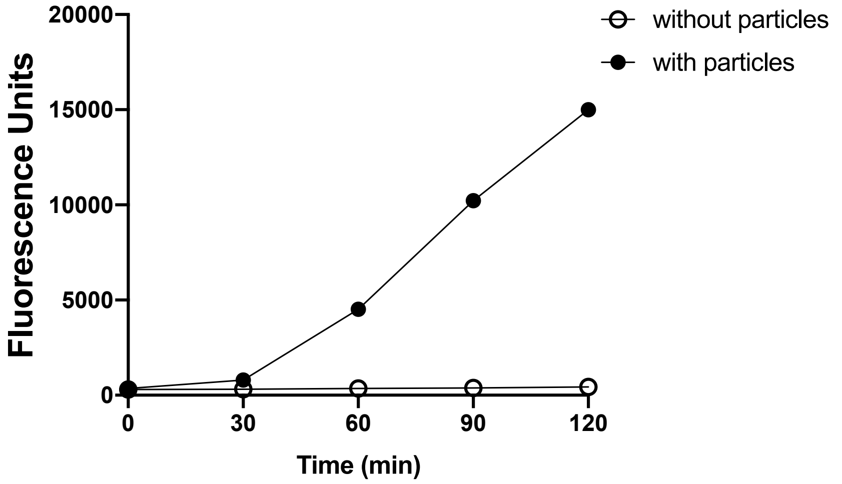

Figure S3. GFP expression profile after ABD2031 infection in *S. aureus* NCTC8325.
The infection experiment was set up with GFP-labeled ABD at MOI=1 and incubated at room temperature for 20 minutes. CYGP media was added to the mix and incubated at 37°C (static). The samples were taken out at indicated time points and washed three times and resuspended in PBS. The fluorescence readings were taken at 480/509nm using BioTek Synergy H1 Hybrid plate reader.
