# Supplementary figures and images for: Dynamics of Antibacterial Drone Establishment in Staphylococcus aureus: Unexpected Effects of Antibiotic Resistance Genes
Source: mBio. 2021 Nov 16;12(6):e02083-21. doi: 10.1128/mBio.02083-21 (PMC8593670; doi:10.1128/mBio.02083-21)

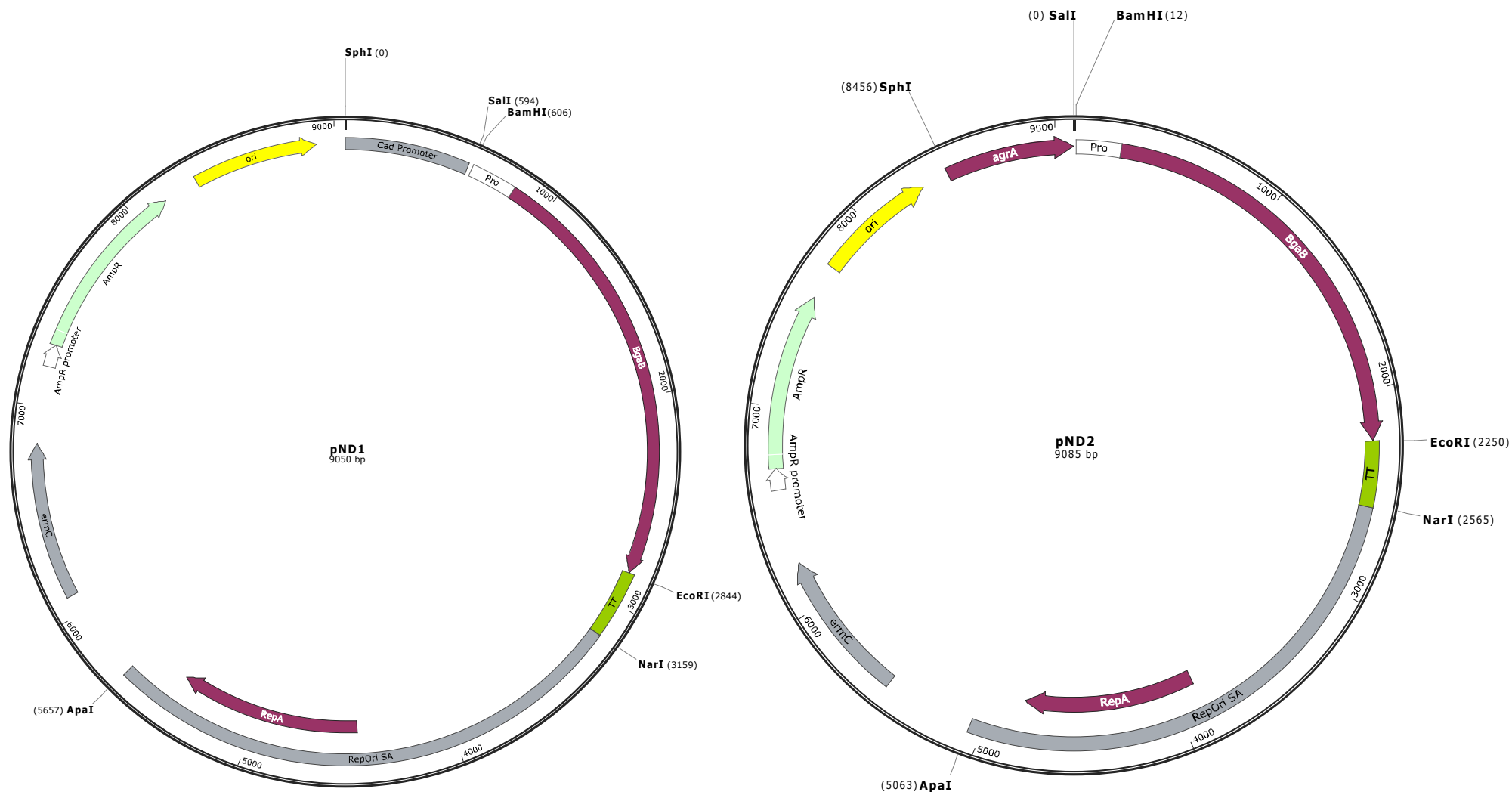

**Figure S4. Plasmid map of plasmid pND1 and pND2.**

Supplement: FIG S4 [file mbio.02083-21-sf004.pdf]
