# Supplementary material for: Dynamics of Antibacterial Drone Establishment in Staphylococcus aureus: Unexpected Effects of Antibiotic Resistance Genes
Source: mBio. 2021 Nov 16;12(6):e02083-21. doi: 10.1128/mBio.02083-21 (PMC8593670; doi:10.1128/mBio.02083-21)
Supplement: FIG S5 [file mbio.02083-21-sf005.pdf]

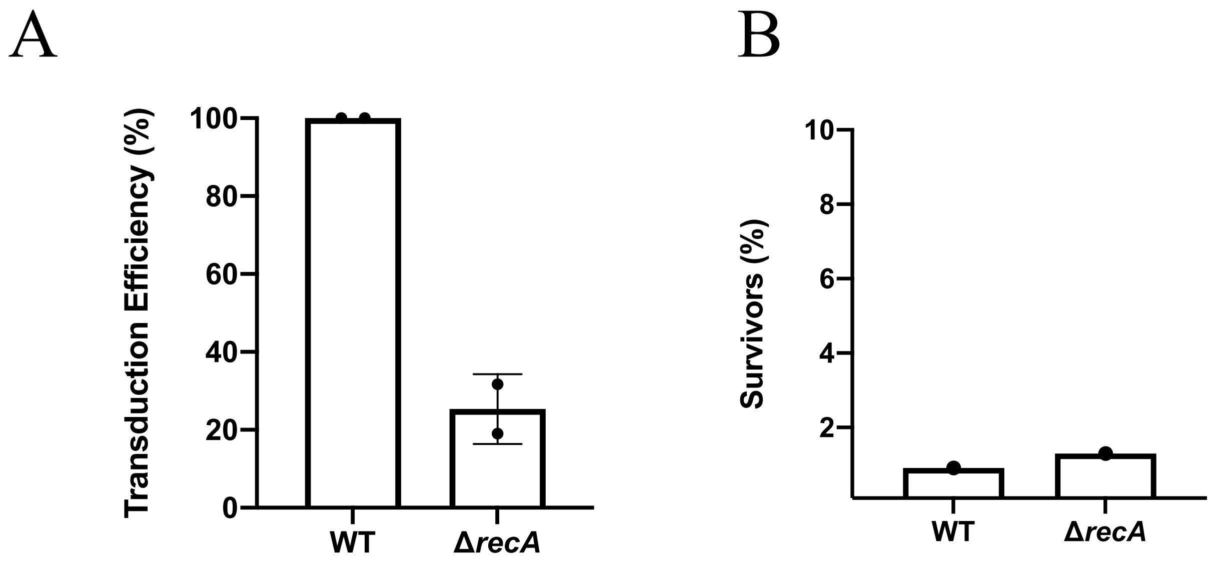

Figure S5. Effect of RecA on ABD transduction and killing efficiencies. (A) Transduction titer was confirmed by mixing equal number of ABD2001 particles with RN450 and RN450 Δ*recA* for 30 minutes and plating on TSB Tc5. Graph represent data from two independent experiments. **(B)** Equal number of ABD2003 particles were mixed with 100µl of cells and survivors were calculated using cfu on TSB agar plates.
