# Supplementary material for: Dynamics of Antibacterial Drone Establishment in Staphylococcus aureus: Unexpected Effects of Antibiotic Resistance Genes
Source: mBio. 2021 Nov 16;12(6):e02083-21. doi: 10.1128/mBio.02083-21 (PMC8593670; doi:10.1128/mBio.02083-21)
Supplement: FIG S6 [file mbio.02083-21-sf006.pdf]

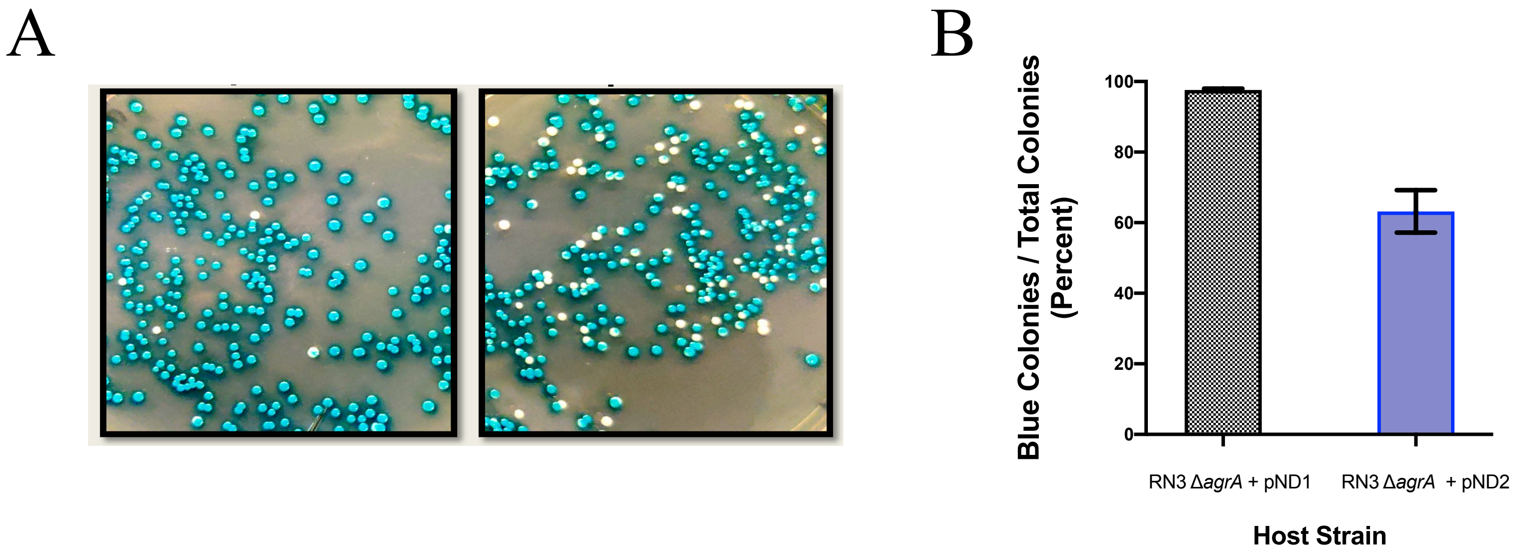

Figure S6. Blue-white colony screening of ABD-infected cells. The RN3 Δ*agrA*+pND1 (**ND58-left image**) and RN3 Δ*agrA*+pND2 (**ND59-right image**) were infected with ABD2016 (*cadA*-tagged ABD2003) (CdR) at MOI-C = 0.3. The transduction titers were calculated in RN3 Δ*agrA* on selection plates GL-Cd100. The infection tubes were incubated at room temperature for 30 minutes, followed by serial dilution and plating of 100µl of 10^{-5} dilution on TSB X-gal. The plates were incubated at 37°C overnight. **(B)** The percentage of blue cells vs. total cells were calculated and correlated with survivors on one-hit killing curve assay.
