# Supplementary material for: Dynamics of Antibacterial Drone Establishment in Staphylococcus aureus: Unexpected Effects of Antibiotic Resistance Genes
Source: mBio. 2021 Nov 16;12(6):e02083-21. doi: 10.1128/mBio.02083-21 (PMC8593670; doi:10.1128/mBio.02083-21)
Supplement: FIG S7 [file mbio.02083-21-sf007.pdf]

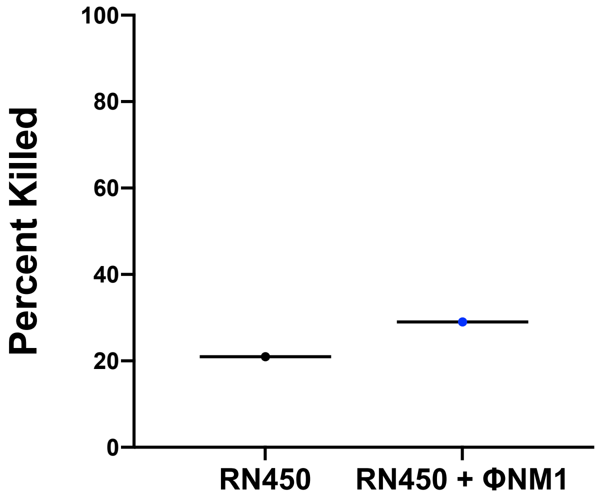

Figure S7- Comparison of ABD killing efficiencies in RN450 and RN450 ϕNM1. Equal number of ABD particles were mixed with 100µl of cells and survivors were calculated using cfu on TSB agar plates.
